# Dynamic Stereopsis Is Abnormal in Treated Anisometropic Amblyopia

**DOI:** 10.1167/iovs.66.14.10

**Published:** 2025-11-04

**Authors:** Yiya Chen, Yao Chen, Robert F. Hess, Jiawei Zhou

**Affiliations:** 1 National Engineering Research Center of Ophthalmology and Optometry, Eye Hospital, Wenzhou Medical University, Wenzhou, People's Republic of China; 2 State Key Laboratory of Eye Health, Eye Hospital, Wenzhou Medical University, Wenzhou, People's Republic of China; 3 Department of Ophthalmology, The First Affiliated Hospital of Anhui Medical University, Hefei, Anhui, People's Republic of China; 4 McGill Vision Research, Department of Ophthalmology and Visual Sciences, McGill University, Montreal, Quebec, Canada

**Keywords:** treated amblyopia, dynamic stereopsis, binocular function, anisometropia, binocular function

## Abstract

**Purpose:**

The purpose of this study was to assess the dynamic stereoscopic function in treated anisometropic amblyopes with restored visual acuity.

**Methods:**

Twelve treated anisometropic amblyopes (best-corrected visual acuity [BCVA] ≤ 0.1 logMAR), 8 non-amblyopic anisometropes, and 12 age-matched emmetropes with normal vision (BCVA ≤ 0.0 logMAR) participated in this experiment. Stimuli comprised 50 randomly positioned, limited-lifetime Gabor elements with lateral motion (0.17 degrees/s to 5.33 degrees/s): half of the elements moving in one direction were presented at the fixation plane and the other half moving in the opposite direction were presented at an uncrossed disparity relative to the fixation plane. Motion directions were counterbalanced across planes, and dynamic stereo performance was quantified using a staircase method.

**Results:**

For the range of motion speeds we tested, we observed clear speed tuning of the stereo sensitivity for all three groups (R_emmetropia_ = 0.93, R_anisometropia_ = 0.97, and R_treated-amblyopia_ = 0.75). Treated amblyopes showed significantly reduced dynamic stereo sensitivity (*P* *=* 0.007) compared to that of other two groups, although speed-tuning shapes did not differ. Dynamic stereopsis sensitivity was uncorrelated with anisometropia degree or static stereopsis.

**Conclusions:**

Even with restored visual acuity, treated amblyopes continues to exhibit binocular deficits, quantified here in terms of dynamic stereopsis. This highlights the need for therapies specifically designed to restore binocular visual function for a comprehensive cure.

Amblyopia, commonly referred to as “lazy eye,” is a neurodevelopmental visual disorder that typically arises in early childhood. It often results from conditions such as strabismus (misaligned eyes), anisometropia (a significant difference in refractive error between the two eyes), visual deprivation (e.g., cataracts), or high bilateral refractive errors.^1^ It is characterized by reduced visual acuity in one or both eyes, despite the absence of any apparent structural abnormalities in the eye itself. In addition to reduced visual acuity, amblyopia has been associated with deficits in a range of monocular and binocular visual functions, including contrast sensitivity, binocular balance, stereopsis, global processing, and visuomotor behavior, among others.^2^^–^^9^ Patching is considered the gold standard for amblyopia treatment. According to the Amblyopia Preferred Practice Pattern guidelines, patients can benefit from the patching therapy even beyond the critical period, particularly if they have not previously been treated.^1^ This involves covering the fellow eye with a patch for several hours per day, which is designed to “force” the brain to rely on the amblyopic eye. Over time, this can strengthen the visual pathway of the amblyopic eye and improve its visual acuity. However, studies have found that even after long-term patching therapy has restored visual acuity in the amblyopic eye, amblyopes continue to exhibit abnormalities in spatial and temporal visual perception. For instance, anisometropic and strabismic amblyopes who have achieved normal visual acuity after patching still show reduced monocular contrast sensitivity at moderate to high spatial frequencies.^10^ Studies have also demonstrated that “treated” amblyopes exhibit imbalanced binocular perception across a wide range of spatial frequencies.^11^^–^^13^ Chen et al. (2023)^14^ reported that “treated” anisometropic amblyopes had significantly higher thresholds for simultaneous temporal discrimination under dichoptic conditions compared with healthy controls. These findings indicate that functional deficits associated with amblyopia whose processing site is in the early stage of visual information processing (i.e. V1), persist even after clinical letter acuity has been normalized through patching therapy.

Stereopsis, a higher-level binocular visual function, is essential for discriminating three-dimensional spatial relationships in visual scenes, which are critical for everyday interactions. Impairment of stereo vision is one of the most common binocular functional abnormalities in amblyopia.^15^^,^^16^ Although some studies have shown that stereoacuity improves with improved visual acuity after patching or dichoptic therapy,^17^^,^^18^ deficits in stereo vision are not fully restored in treated amblyopes.^18^^–^^20^ Recent studies reported that nearly $\frac{2}{3}$ of refractive amblyopes whose visual acuity had been restored to normal levels had residual abnormal static stereopsis.^20^^,^^21^ However, dynamic stereo plays a more prominent role in our daily lives. Studies have shown that the ability to discriminate dynamic stereoscopic targets may differ from that of static stereoscopic targets.^22^^–^^24^ Evidence suggests that patients with strabismic amblyopia diagnosed as stereo-blind based on static disparity-based random dot test may be able to respond to dynamic disparity stimuli,^25^^–^^27^ indicating that their stereoscopic function is not completely lost. Moreover, the visual processing sites for static and dynamic stereopsis appear to differ where the static stereopsis is primarily processed in early cortical areas,^28^ whereas dynamic stereopsis is likely to be processed at later stages of the cortical pathway.^29^^–^^31^ Furthermore, it is now recognized that the critical period for visual development is longer for neural mechanisms at later stages of the cortical pathway^32^^,^^33^ and, thus, given the information above, there is every reason to expect different effects for static and dynamic stereopsis in a developmental condition such as amblyopia. Investigating the performance of dynamic stereopsis in treated amblyopic patients whose visual acuity has been restored to normal levels cannot only provide a more comprehensive understanding of the deficits of amblyopia, particularly those involving sites at higher-level in the visual pathway, but can also offer new evidence for defining the treatment endpoint in amblyopia.

A psychophysical paradigm involving disparity and lateral motion processing was developed in our previous study, which eliminated monocular cues, allowed adjustment of stimulus spatial frequency, motion speed, and direction, and presented disparities at subpixel levels. Using this paradigm, we found that human dynamic stereo perception is tuned to the speed of lateral motion.^29^ In the present study, we applied this psychophysical paradigm to quantitatively assess dynamic stereopsis in adults who had been successfully treated in terms of their monocular acuity deficit. Given that anisometropia itself may cause impairment in static stereo vision,^34^^,^^35^ we additionally included a control group of anisometropic subjects without a history of amblyopia, matched to the “treated” amblyopic group by the degree of anisometropia. We found that dynamic stereopsis in treated amblyopes depended on the speed of the elements in depth and that dynamic stereo sensitivity was significantly lower than in healthy non-amblyopic emmetropic and anisometropic controls. These results also suggest that dynamic stereoscopic measurements can play an important role in evaluating the higher-level visual deficits in amblyopia.

## Methods

### Participants

Twelve treated anisometropic amblyopes (average age = 23.61 ± 3.00 years old, individual clinical characteristics see the Table), 8 non-amblyopic anisometropes (average age = 22.22 ± 2.57 years old, individual clinical characteristics see Supplementary Table S1) and 12 non-amblyopic emmetropes (average age = 23.38 ± 2.81 years old) participated in our experiment. The basic demographic information of each participant, including age, sex, and refractive status was obtained at their first visit. All participants underwent comprehensive ophthalmic examinations, including best-corrected visual acuity (BCVA), subjective refraction, slit-lamp biomicroscopy, fundus examination, and static stereopsis test (assessed using Yan's Glasses-free Randot Stereotest, China). All subjects had normal or corrected to normal visual acuity. Several criteria were used to recruit the treated anisometropic amblyopes: (1) a definite prior diagnosis of anisometropic amblyopia based on interocular visual acuity difference of two or more lines (with the better eye within the normal range), presence of anisometropia, and absence of other structural eye diseases^1^; (2) a history of amblyopia treatment; (3) BCVA ≤ 0.1 logMAR in the previously amblyopic eye and BCVA ≤ 0.0 logMAR in the previously fellow eye; (4) a visual acuity difference between the previously amblyopic eye and fellow eye ≤ 0.2 logMAR; and (5) maintenance of normal visual acuity in the previously amblyopic eye at least 3 months after treatment.^14^ For non-amblyopic anisometropes, the inclusion criteria were: (1) interocular refractive error > 2 Diopter, and (2) BCVA ≤ 0.0 logMAR in both eyes. Emmetropes were required to meet the following: (1) equivalent spherical lenses between −0.5 and +0.75 Diopter and (2) uncorrected visual acuity ≤ 0.0 logMAR. The hole-in-the-card tests were used to measure the motor dominant eye in the emmetropes and the non-amblyopic anisometropes.^36^ Prior to stereoacuity assessment, all participants underwent subjective refraction to ensure attainment of BCVA. Trial frames were provided when habitual optical corrections were inadequate for achieving optimal visual acuity. We obtained signed consent forms from each subject prior to the experiment. This study was approved by the Ethics Committee at Wenzhou Medical University and adhered to the tenets of the Declaration of Helsinki.

**Table.** Clinical Characteristics of the Treated Amblyopes

Subject	Sex/Age	Refraction OD/OS	BCVA (logMAR) OD/OS	Randot Stereo Acuity (Arc Sec)	History	BCVA Pretreatment (logMAR) OD/OS
A1	F/23	+1.0 DS-0.5 DC × 70^*^ −2.5 DS	0^*^ −0.1	400	Detected at 9 y old; received glasses, patching, bead-threading, red filter treatment; visual acuity recovered to normal at 10 y old	0.4^*^ 0
A2	F/22	0.75 DS^*^ −3.25 DS	0^*^ 0	200	Detected at 7 y old; received glasses, patching, bead-threading; visual acuity recovered to normal at 10 y old	≥ 2 lines
A3	M/29	+2.75 DS-5.0 DC × 20^*^ −3.75 DS-1.0 DC × 177	0^*^ −0.1	200	Detected at 15 y old; received glasses; visual acuity recovered to normal at 17 y old	0.2^*^ 0
A4	M/24	−4.0 DS −0.25 DS^*^	−0.1 0^*^	60	Detected at 4 y old; received glasses, patching, bead-threading; visual acuity recovered to normal at 8 y old	0 0.3^*^
A5	F/28	−4.0 DS +1.50 DS^*^	0 0.1^*^	80	Detected at 5 y old; received glasses, patching, bead-threading, strabismus surgery at 26 y of age; visual acuity recovered to normal at 24 y old	0 0.8^*^
A6	M/22	+3.0 DS-2.0 DC × 170^*^ +0.75 DS-0.75 DC × 170	0^*^ −0.1	400	Detected at 11 y old; received glasses, patching, red filter treatment; visual acuity recovered to normal at 11 y old	0.8^*^ 0
A7	M/22	+4.25 DS-1.5 DC × 25^*^ −0.75 DS-0.75 DC × 170	0^*^ 0	60	Detected at 3 y old received glasses, patching, bead-threading; red filter treatment; visual acuity recovered to normal at 6 y old	0.5^*^ 0.2
A8	F/28	+1.75 DS-0.25 DC × 111 +3.25 DS-0.75 DC × 24^*^	−0.1 −0.1^*^	60	Detected at 5 y old; received glasses, patching; visual acuity recovered to normal at 10 y old	≥ 2 lines
A9	F/19	−1.25 DS-1.0 DC × 72 +2.5 DS-1.5 DC × 163^*^	0 0.1^*^	200	Detected at 10 y old; received glasses; visual acuity recovered to normal at 14 y old	0 0.3^*^
A10	F/20	−1.25 DS-0.5 DC × 106 +3.5 DS-1.25 DC × 22^*^	−0.1 0^*^	200	Detected at 5 y old; received glasses, patching, bead-threading, red filter treatment, atropine; visual acuity recovered to normal at 8 y old	0 0.3^*^
A11	F/24	−2.75 DS^*^ −4.75 DS-0.5 DC × 10	0^*^ 0	60	Detected at 5 y old; received glasses, patching, bead-threading; red filter treatment; visual acuity recovered to normal at 10 y old	0.2^*^ 0
A12	F/23	−5.25 DS-1.0 DC × 12^*^ −2.0 DS-0.25 DC × 170	0^*^ −0.1	40	Detected at 6 y old; received glasses, patching, bead-threading; visual acuity recovered to normal at 11 y old	0.8^*^ 0

BCVA, best-corrected visual acuity; F, female; logMAR, logarithm of the minimum angle of resolution; M, male; OD, right eye; OS, left eye; Randot Stereo acuity, measure of stereopsis by Randot stereo test in arc seconds.

* Pre-diagnosed amblyopic eye; For two of the twelve treated amblyopic patients, historical pretreatment acuity records were unavailable; however, both subjects confirmed ≥0.2 logMAR interocular acuity differences at treatment initiation.

### Apparatus

The experiment was programmed with MATLAB R2016a (MathWorks, Natick, MA, USA) and PsychToolBox version 3.0.14 extension and conducted in a dark room at a viewing distance of 171 cm. All stimuli were presented on a gamma-corrected LG D2792PB 3D LED screen (LG Life Sciences, Seoul, Korea) with a resolution of 1920 × 1080 pixels and a refresh rate of 60 hertz (Hz). We used the Bits^#^ stimulus processor (Cambridge Research Systems Ltd., Rochester, UK) to produce contrast resolution of 14-bit, and used polarized glasses to dichoptically present stimuli to the observers throughout the psychophysical experiments. The average luminance through the polarized glasses was 36.5 cd/m^2^.

### Stimuli

**Figure 1.**
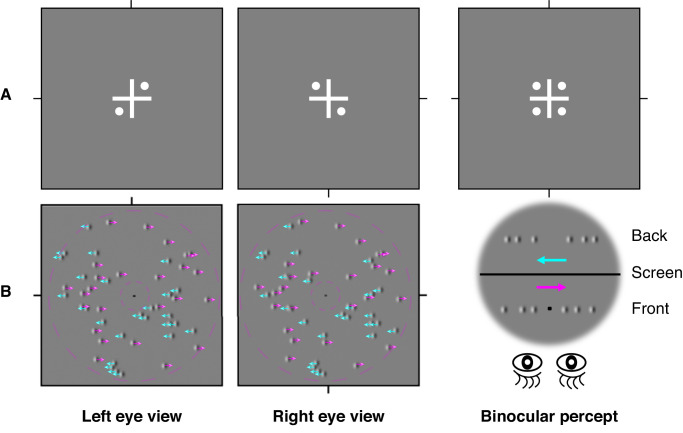
**Illustration of the experimental design.** (**A**) Binocular alignment task. Subjects were asked to adjust the positions of four white dots to achieve equal inter-dot spacing. (**B**) An illustration of the laterally moving stimuli in the dynamic stereoscopic measurement. Two depth planes: one plane was at the fixation plane and the other at an uncrossed disparity plane relative to the fixation plane. Each plane contained 25 horizontally moving Gabors (leftward/rightward), randomly positioned within the display (diameter = 6.15 degrees in visual angle), while avoiding a central fixation exclusion zone (diameter = 1.11 degrees in visual angle). The Gabor envelope and sinewave carrier moved together at the same speed. Subjects were asked to indicate the perceived motion direction (either left or right, as indicated by the *red* or *blue arrows* separately) of Gabors in the front fixation plane.

A similar psychophysical paradigm as Chen et al. (2022)^29^ was used in this experiment to measure dynamic stereopsis across three groups of observers. Specifically, we used a Gabor array consisting of an equal number of Gabors moving in one of two opposite directions (Fig. 1) to measure stereoacuities at six different velocities (0.17 degrees/s, 0.33 degrees/s, 0.67 degrees/s, 1.33 degrees/s, 2.67 degrees/s, and 5.33 degrees/s). The stereoacuities at different speeds were measured in a random order. The Gabor array contained 50 Gabor elements, each with a spatial frequency of 3 cycles/degree and a size of one cycle. During the experiment, the envelope and sinewave carrier of each Gabor element were moved together at the same speed. Two depth planes were included with one at the fixation plane and the other at an uncrossed disparity plane. Each plane contained 25 Gabors moving horizontally either leftward or rightward (see Fig. 1). Gabors assigned to the same depth plane were always shifted in the same direction. We applied a Gaussian window to each Gabor element and recalculated the peak of the Gaussian function to obtain the subpixel interpolation of the stimuli, thereby enabling a higher precision measurement of stereoacuity (i.e. smaller disparity between two depth planes). The presentation duration of the two depth planes was set to 1000 ms. Subjects were instructed to report the direction of motion (left or right) of the Gabor elements in the front fixation plane. To minimize the possibility of subjects tracking individual Gabor elements, the Gabors were shown to each eye at random positions within a display area of 6.15 degrees in diameter (in visual angle). Each Gabor had a 5% probability of limited lifetime in each presentation frame. Gabor elements that reached the limited lifetime would reappear at a random location within the display window. Similarly, Gabors moving to the edge of the display window would disappear and immediately reappear at the opposite edge. Furthermore, to facilitate fixation control, we set an empty space around the central fixation point (diameter = 1.11 degrees in visual angle).

### Procedure

Prior to the formal experiments, all subjects completed practice trials to familiarize themselves with the task. A minimum performance criterion of 90% correct rate at 1503 arc seconds disparity (the largest disparity we set) was required. In all experimental sessions, subjects first performed an alignment task (see Fig. 1A), in which they adjusted the positions of four white points so that the distances between adjacent points were equal. This ensured proper binocular alignment and fusion. No suppression was reported by any subject during the alignment procedure. After that, a dichoptic viewing frame with the fixation point at the center of the display was presented before each trial and remained visible through the entire visual task to help maintain eye alignment via peripheral fusion. We displayed Gabors in two depth planes (a fixation plane and an uncrossed disparity plane relative to the fixation plane) for a duration of 1000 ms. Subjects were asked to report whether the Gabor elements in the front fixation plane moved to the right or left without time limitation (see Fig. 1B). The next trial began 200 ms after a response was given. A 3-down-1-up staircase procedure was used to determine the minimum uncrossed disparity (i.e. D_min_) at which subjects could discriminate the depth planes defined by Gabors moving in different directions. The disparity for the initial trial was set at 40 pixels (i.e. equivalent to 1503 arc seconds). Disparity was adjusted trial-by-trial based on the subject’s performance: the step size was set as 50% prior to the first reversal and 20% thereafter. The staircase terminated after the sixth reversal. We used the average of the last five reversals to calculate the threshold and variance. To improve accuracy, measurements were repeated 3 times at each speed, resulting in a total of 15 reversals used to determine the stereoacuity for each condition. We provided different auditory feedback for correct and incorrect answers.

### Data Analysis

We recorded stereo thresholds in pixel units and converted them to stereo sensitivity in arc seconds (i.e. the reciprocal of stereo threshold = 1/D_min_). Two-way repeated measures analysis of variance (ANOVA) was used to examine differences in stereo sensitivity across speeds and among groups. All statistical analyses were performed using IBM-SPSS 26.0 (IBM Inc., Armonk, NY, USA). Referring to previous studies^37^^,^^38^ that fitted speed tuning curves to cellular responses in the middle temporal (MT) area of the macaque visual cortex, the current study used a logarithmic Gaussian function to fit the speed tuning function for stereo sensitivities with the following equation:

$$\begin{array}{c} \varphi =\varphi_{0}+A\times exp\left[-\frac{1}{2\times \sigma^{2}}{\left(log\frac{s}{s_{p}}\right)}^{2}\right] \end{array}$$

where *φ* represents the stereo sensitivity, and *s* is the stimulus speed in degrees per second. There are four free parameters in the function: *φ*_0_ means a general amplitude, *A* is the peak amplitude, σ is the (logarithmic) tuning width, and *S_p_* is the preferred speed.

## Results

### Dynamic Stereo Visual Performance of Three Groups

**Figure 2.**
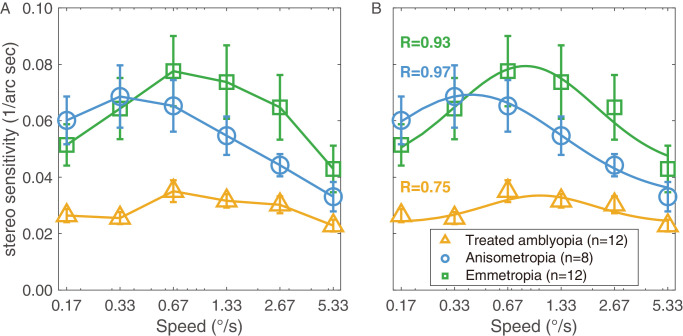
**The speed-tuning function of dynamic stereo sensitivity.** (**A**) The stereo sensitivity as a function of the speed of laterally moving Gabor elements for three groups (treated anisometropic amblyopes, non-amblyopic anisometropes, and non-amblyopic emmetropes). (**B**) The fitted speed tuning curves of stereo sensitivity for three groups. R indicates the goodness-of-fit of each group, respectively. The *yellow triangle symbols* represent the treated amblyopic group, the *green square symbols* represent the emmetropic group, and the *blue circle symbols* represent the non-amblyopic anisometropic group. The error bars represent the standard error (SE).

To illustrate whether speed affects performance in our stereoscopic task, we measured the stereo sensitivity of all subjects across six speeds of Gabor movement (0.17 degrees/s, 0.33 degrees/s, 0.67 degrees/s, 1.33 degrees/s, 2.67 degrees/s, and 5.33 degrees/s) at a Gabor spatial frequency of three cycles/degree. Figure 2A shows the stereo sensitivity curves for the three participant groups as a function of Gabor speed. The bell-shaped tuning curves indicate that stereo sensitivity is speed-tuned, with peaks occurring between 0.33 degrees/s and 1.33 degrees/s, which is consistent with previous findings.^29^ To compare whether stereo sensitivity differed among the three groups, we applied a two-way repeated measures ANOVA (between-subject factor: group and within-subject factor: speed) and showed that the stereo sensitivities were significantly different across different speeds: [F (3.495, 101.359) = 15.635, *P* < 0.001] and among different groups [F (2, 29) = 5.998, *P* *=* 0.007]. The interaction effect between speed and group was significant [F (10, 145) = 3.979, *P* < 0.001). Post hoc analysis with least significant difference (LSD) correction revealed significant differences in stereo sensitivity between the treated amblyopic group and the non-amblyopic anisometropic group (*P* *=* 0.030), as well as between the treated amblyopic group and the emmetropic group (*P* *=* 0.002). Although the difference between the non-amblyopic anisometropic group and the emmetropic group was not significant (*P* *=* 0.478).

A bell-shaped tuning of stereo sensitivity as a function of Gabor speed was found in all groups. We therefore fitted the mean stereo sensitivity for each group separately using a logarithmic Gaussian model that has been used in previous studies to model speed tuning curves of cells in the middle temporal region of macaque visual cortex.^37^^,^^38^ The model successfully captured the average data for all three groups (Fig. 2B; R_emmetropia_ = 0.93, R_anisometropia_ = 0.97, and R_treated amblyopia_ = 0.75), suggesting that dynamic stereopsis is speed-tuned across all three groups.

**Figure 3.**
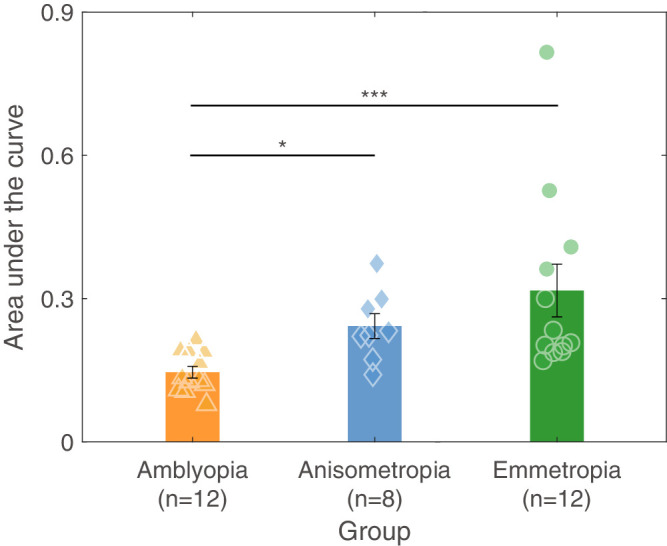
**Area under the curve defining dynamic stereo sensitivity in three groups.** The *green bars* and *dots* represent the emmetropic group; the *blue bars* and *diamonds* represent the non-amblyopic anisometropic group; the *yellow bars* and *triangles* represent the treated amblyopic group. * Represents *P* ≤ 0.05, *** represents *P* ≤ 0.001. Error lines represent SE.

To further evaluate differences in dynamic stereopsis among the three groups, we calculated the area under the curve (AUC) that defines the relationship between stereo sensitivity and lateral motion speed for each group (Fig. 3). The Kruskal-Wallis H test indicated a statistically difference in AUC among the three groups (H = 14.336, *P* *=* 0.001). Post hoc analysis revealed statistically significant differences between the treated amblyopic group and the emmetropic group (H = −13.417, *P* *=* 0.001), as well as between the treated amblyopic group and the non-amblyopic anisometropic group (H = −12.208, *P* *=* 0.013), whereas no significant difference was observed between the non-amblyopic anisometropic and the emmetropic groups (H = 1.208, *P* *=* 1.000). The results suggested that the treated amblyopic group had poorer performance in dynamic stereopsis compared with the other two groups.

To assess whether the shape of the speed tuning function differed among groups, we calculated the peak (i.e. preferred speed) and bandwidth of the tuning function for each group. A one-way ANOVA revealed that there was no significant difference among the three groups in either preferred velocity [F (2, 29) = 1.918, *P* *=* 0.165] or bandwidth [F (2, 29) = 1.401, *P* *=* 0.262].

### The Correlation Among Degree of Anisometropia, Static Stereopsis, and Dynamic Stereopsis

Visual acuity in both the fellow/dominant eye and the amblyopic/nondominant eye showed no significant difference among the three groups [fellow/dominant eye: F (2, 29) = 2.942, *P* *=* 0.069, amblyopic/nondominant eye: F (2, 29) = 1.812, *P* *=* 0.181, 1-way ANOVA]. No significant correlation has been found between the BCVA of the amblyopic eye and dynamic stereopsis in treated amblyoipa (see Supplementary Figure S1). Static stereopsis, as measured by Randot Stereotest, was distinctly different among the three groups [F (2, 18.873) = 11.870, *P* < 0.001, Welch test], where the treated amblyopic group was significantly worse than other two groups (versus the non-amblyopic anisometropic group: *P* *=* 0.001, versus the emmetropic group: *P* < 0.001, post hoc test with Games-Howell correction). The degree of anisometropia was 3.56 ± 0.35 Diopter (mean ± SE) in the treated amblyopic group, 2.89 ± 0.17 Diopter in the non-amblyopic anisometropic group, and 0.52 ± 0.33 Diopter in the emmetropic group. A Welch test suggested significant difference among the three groups [F (2, 15.187) = 81.312, *P* < 0.001]. Post hoc analysis with Games-Howell correction showed no statistical difference between the treated amblyopic group and the non-amblyopic anisometropic group (*P* *=* 0.264).

**Figure 4.**
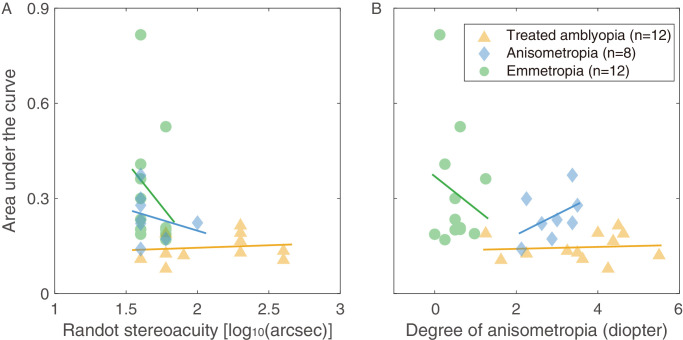
**The correlation** **among** **degree of anisometropia, static stereopsis,** **and dynamic stereopsis.** (**A**) The correlation between the static stereopsis (Randot stereoacuity) and dynamic stereopsis for three groups. (**B**) The correlation between the degree of anisometropia and dynamic stereopsis for three groups. The *yellow triangle symbols* represent the treated amblyopic group, the *green square symbols* represent the emmetropic group, and the *blue circle symbols* represent the non-amblyopic anisometropic group.

To investigate whether dynamic stereopsis was corelated with static stereopsis, we performed a two-tailed Pearson's correlation test between the AUC of dynamic stereo sensitivity and Randot Stereoacuity in the treated amblyopic group, which suggested no significant correlation (*r* = 0.130, *P* *=* 0.688; Fig. 4a). Spearman's correlation tests indicated no significant correlation in the other two control groups either (anisometropic group: rho = −0.343, *P* *=* 0.406, and the emmetropic group: rho = −0.367, *P* *=* 0.240).

To investigate whether dynamic stereopsis was associated with the degree of anisometropia, we performed a two-tailed Pearson's correlation test for the treated amblyopic and non-amblyopic anisometropic groups, and a Spearman's correlation test for the emmetropic group. The result showed no significant correlation between the AUC of dynamic stereo sensitivity and the degree of anisometropia in any group (treated amblyopic group: *r* = 0.092, *P* = 0.776; non-amblyopic anisometropic group: *r* = 0.458, *P* *=* 0.254, and the emmetropic group: rho = 0.075, *P* *=* 0.818; Fig. 4b).

## Discussion

In the present study, we used a novel psychophysical paradigm previously developed for individuals with normal vision,^29^ which involves two depth planes with elements moving laterally in opposite directions. We evaluated dynamic stereopsis in three groups: the non-amblyopic emmetropic group, the non-amblyopic anisometropic group, and the treated anisometropic amblyopic group. The results demonstrated that stereopsis was tuned to stimulus speed in all three groups. In addition, the results showed that treated amblyopes exhibited significantly worse dynamic stereo performance compared with both emmetropic and non-amblyopic anisometropic controls. Similar to our previous study using the same dynamic stereopsis paradigm,^29^ we measured stereo sensitivity for lateral motion stimuli at 6 speeds ranging from 0.17 degrees/s to 5.33 degrees/s. Previous studies have reported that most MT lobe (area MT/V5) neurons were tuned to slower speeds, typically in the range of 0 degrees/s to 10 degrees/s.^37^^,^^39^ The current results revealed similar bell-shaped curve for stereo sensitivity across lateral motion speeds (see Fig. 2B), with peaks occurring within a relatively low velocity range (between 0.33 degrees/s and 1.33 degrees/s).

One conclusion of our study is that stereopsis, in the present case dynamic stereopsis, can be impaired in amblyopes even after the amblyopic eye's visual acuity has been restored to normal levels through patching treatment. Our findings indicate that this deficit is attributable not to the presently corrected anisometropia but to the previously treated amblyopia. Currently, clinical practice focuses on improving the monocular visual acuity of the amblyopic eye as the treatment endpoint. Whereas prioritizing visual acuity recovery is undoubtedly reasonable, this represents neither a comprehensive nor a functionally important remedy. Recovery of binocular function, which is arguably of more functional importance is often defective in treated amblyopes.^40^ Although our research cannot determine whether the impairment in dynamic stereopsis results from amblyopia itself or the treatment process, it adds further weight to the conclusions of previous studies that “treated” amblyopic patients remained deficient in monocular and binocular visual functions,^10^^,^^11^^,^^14^ showing that fully recovered monocular vision in the amblyopic eye does not preclude the existence of other monocular and binocular deficits.

One concern is that suppression in the treated amblyopia may have played a role in the dynamic stereoacuity deficits. Webber et al. (2020) reported that suppression, rather than visual acuity loss, limits stereoacuity in observers with abnormal vision development.^41^ Treated amblyopes have been found to exhibit abnormal interocular suppression, leading to unbalanced binocular vision and an elevated dichoptic temporal synchrony threshold.^11^^,^^14^ Further studies using conditions that balance interocular contrast based on their suppression status are needed to address the question.

Another possible explanatory factor involves residual oculomotor dysfunction in treated amblyopes. Studies have shown that fixation instability, increased saccade latency, and poor binocular coordination of fixation in amblyopia may not be fully restored by patching treatment.^42^^,^^43^ Whereas Zhou et al. (2022) suggested that early and efficient intervention for amblyopia could additionally restore eye movement functions.^44^ Our previous studies suggested that eye movements did not significantly influence stereo-speed tuning characteristics.^29^ Therefore, whereas persistent oculomotor abnormalities may plausibly elevate dynamic stereo thresholds in treated amblyopia, their specific contribution to stereo-speed tuning parameters requires further investigation.

Whereas auditory feedback is a standard component of psychophysical paradigms (where performance differences between feedback/no-feedback conditions may occur), we maintain that our intergroup comparisons remain valid as all participants underwent identical testing protocols. Regarding amblyopia-specific considerations, current literature suggests potential audiovisual integration deficits,^45^ although whether these persist post-treatment remains unclear. Importantly, our auditory cues served solely as response indicators rather than integrated stimulus components – a methodological distinction compared with audiovisual integration studies. Furthermore, all participants received comprehensive task training to ensure full familiarity with the testing procedure. Therefore, we believe auditory cues minimally impacted our core findings.

Whereas our findings consistently demonstrated that treated amblyopic patients exhibited poorer dynamic stereopsis compared with non-amblyopic anisometropes and emmetropes, the modest sample size does constrain our ability to perform more detailed subgroup analyses. Specifically, treatment during versus after the critical period of visual plasticity might contribute to the observed stereoacuity deficits. We accordingly have subdivided the treated amblyopes into two subgroups according to the age when they were diagnosed with amblyopia (<7 years old and ≥7 years old). We found a significant difference in static stereopsis between two subgroups (*P* = 0.005, Mann-Whitney *U* test), but no significant difference in dynamic stereopsis (*P* = 0.794, Two-sample Independent T test). However, a larger sample size would provide more statistical power to enable a definitive conclusion. Additionally, potential differences in the speed-tuning characteristics of dynamic stereopsis among amblyopia subtypes could not be adequately investigated due to limited statistical power.

It is likely, based on the current physiology, that static and dynamic stereopsis are processed at different stages along the cortical pathway. The site of static stereopsis has been identified in early cortical areas such as V2/V3^28^ whereas dynamic stereopsis of the type measured here with limited-lifetime motion stimuli, is likely to be processes at later stages in the cortical pathway, for example, area MT where cells are tuned for both motion and stereo.^29^^–^^31^ What is the nature of the deficit underlying the current results? There are a number of possibilities: consequences of a downstream deficit or a deficit located in higher reaches of the cortical pathway involving the extraction of disparity from noisy (in this case, motion) stimuli. Both explanations are plausible. Monkey studies have shown strong suppressive responses in areas V2/V3 of amblyopic animals, and human studies have similarly demonstrated a strong relationship between the loss of static stereopsis and the strength of suppression.^41^ A low-level, suppression-based explanation could also account for our results using dynamic stimuli. However, an argument against a common, low-level cause is that we did not find any correlation between the deficits for static and dynamic stereopsis in treated anisometropic amblyopes. Another explanation, one specific to the site where dynamic stereopsis is processed, may involve either a purely temporally based deficit, such as processing delays in the amblyopic cortical pathway,^46^^–^^49^ or an issue with extracting signal from noise,^50^^,^^51^ both deficits are known to be present in amblyopia. Furthermore, it remains a possibility that the methodological differences between static and dynamic stereoacuity tests may also contribute to the observed dissociation.

Consequently, this study provides additional evidence for residual deficits in amblyopes whose visual acuity has been restored using patching treatment. This residual binocular deficit involving dynamic stereopsis is likely to be functionally important to these patients.

## Supplementary Material

Supplement 1
