# Supplementary material for: Dynamic Stereopsis Is Abnormal in Treated Anisometropic Amblyopia
Source: Invest Ophthalmol Vis Sci. 2025 Nov 4;66(14):10. doi: 10.1167/iovs.66.14.10 (PMC12598826; doi:10.1167/iovs.66.14.10)
Supplement: Supplement 1 [file iovs-66-14-10_s001.pdf]

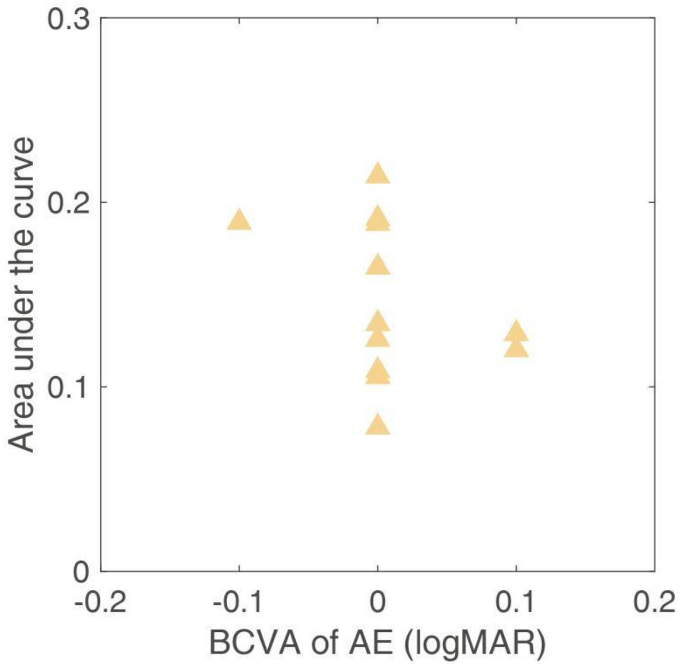

Figure S1. The correlation between best corrected visual acuity of amblyopic eye and dynamic stereopsis.

Table S1. Clinical characteristics of the nonamblyopic anisometropes

Subject	Sex/Age	Refraction OD/OS	BCVA (logMAR) OD/OS	Randot Stereo acuity (Arc sec)
S1	F/24	-4.25DS-0.75DC×180<br>-1DS-0.25DC×145	0<br>0	40
S2	M/25	-4.25DS-0.75DC×19<br>-1.75DS-0.5DC×168	0<br>0	40
S3	F/25	-3.25DS<br>-1DS	0<br>0	40
S4	F/24	-2.75DS-0.25DC×174<br>-0.75DS	0<br>0	40
S5	F/23	-2.25DS-1.25DC×170<br>+0.5DS	0<br>0	100
S6	F/19	+3DS-0.75DC×120<br>-0.25DS-0.25DC×150	0<br>0	40
S7	M/18	-3DS-0.5DC×7<br>-0.25DS-0.25DC×136	-0.1<br>-0.1	60
S8	M/20	-3DS-0.75DC×141<br>0.25DS-0.25DC×8	-0.1<br>-0.1	40

Abbreviations: BCVA, best-corrected visual acuity; F, female; logMAR, logarithm of the minimum angle of resolution; M, male; OD, right eye; OS, left eye; Randot Stereo acuity, measure of stereopsis by randot stereotest in arc seconds
